# An assessment of household water quality among Peace Corps volunteers in Guatemala

**DOI:** 10.1186/s40794-019-0078-8

**Published:** 2019-03-12

**Authors:** Daniel E. Murphy, Scott A. Poe, Jennifer L. Murphy, Rennie W. Ferguson, Susan J. Henderson, Paul Jung

**Affiliations:** 1 Office of Health Services, Peace Corps, 1111 20th Street NW, Washington, DC, 20526 USA; 2 Waterborne Disease Prevention Branch, Division of Foodborne, Waterborne, and Environmental Diseases, Centers for Disease Control and Prevention, 1600 Clifton Road, Atlanta, GA 30329 USA; 3 Division of Parasitic Diseases and Malaria, Center for Global Health, Centers for Disease Control and Prevention, Atlanta GA. Previously Office of Health Services, Peace Corps, Washington DC, USA; 4 Previously Office of Health Services, Peace Corps, 1111 20th Street NW, Washington, DC, 20526 USA

**Keywords:** Peace Corps Volunteers, Traveler, Gastrointestinal illness, Drinking water, Guatemala

## Abstract

**Background:**

Gastrointestinal (GI) illness is the most commonly reported health concern among Peace Corps Volunteers (PCVs) serving in Guatemala. This project identified water types and treatment and storage practices used by PCVs and measured select water quality parameters in their household water.

**Methods:**

A survey about water types and practices was conducted of PCVs in Guatemala. The water type most frequently consumed in the household (“primary drinking water”) and other water types present in the household (“secondary water”) were tested for free chlorine residual (FCR) and for the presence of *Escherichia coli* and total coliforms. A negative binomial regression model was used to analyze data on incidence of self-reported GI illness.

**Results:**

*Tambo* (commercially purified water in a 5-gal bottle) was the water type most frequently (64%) reported as primary drinking water in 39 PCV households. Most (74%) PCVs reported drinking water other than primary drinking water ≥1 day per week; the incidence rate of GI illness per PCV per month was significantly lower among PCVs who reported never consuming water other than primary drinking water compared to those who did (0.4 and 1.6 GI illnesses per PCV per month, respectively) (*p* < 0.05). *E. coli* was not detected in any primary drinking water sample, but was detected in 35% of secondary water samples. Total coliforms were detected in more than two-thirds of primary drinking water and secondary water samples. Nearly all water samples had an FCR of < 0.2 mg/L.

**Conclusions:**

Consuming primary drinking water exclusively likely contributes to reducing the rate of GI illness among PCVs. However, most PCVs reported drinking multiple water types, which may include contaminated secondary water types in the household. All water intended for consumption, including secondary sources within and outside the household, should be properly treated and safely stored.

## Background

The Peace Corps is a U.S. government agency whose mission is to promote world peace and friendship through service work. Peace Corps Volunteers (PCVs) serve in a community for two years to help interested countries meet their needs for trained men and women, to promote understanding of Americans on the part of peoples served, and to promote understanding of peoples served on the part of Americans [1].

PCVs have served in small- to medium-sized urban and rural communities in Guatemala since 1963. Historically, gastrointestinal (GI) conditions have been the most commonly reported health concern among PCVs serving in Guatemala [2]. In 2016, the rate of GI illness among PCVs in Guatemala was 94% higher than the rate in the Inter-America and Pacific region and 63% higher than the global rate, according to data collected through Peace Corps’ Epidemiologic Surveillance System [2]. GI illness not only represents a considerable health burden for PCVs, but also contributes to absence from work and directly affects a PCV’s service.

Before beginning service, PCVs undergo 10 weeks of intensive training in Guatemala, including standardized modules on safe food and water preparation [3]. Peace Corps Guatemala training emphasizes untreated water may be contaminated and waterborne illness can be reduced by consuming three recommended types of water: commercially-purified water, piped water boiled in the household, or piped water filtered in the household using a sub-micron ceramic or candle filter followed by chlorine treatment. When these methods are not available, four temporary water treatment methods are recommended: iodine tablets, iodine 2% liquid, iodine resin filters, and chlorine bleach without microfiltration [4].

The aims of this project were: 1) to identify water types and treatment practices used by PCVs in Guatemala, and 2) to measure select water quality parameters, including free chlorine residual (FCR), *Escherichia coli* bacteria (*E. coli*), and total coliforms, in household water consumed or used by PCVs. The World Health Organization (WHO) recommends an FCR of ≥0.2 mg/L in drinking water during non-emergency periods [5]. Presence of *E. coli* is used as an indicator of fecal contamination, and therefore of the potential presence of pathogens. WHO recommends that water intended for consumption should contain < 1 *E. coli* per 100 mL [5]. Total coliforms, which include both fecal and environmental bacterial species, are used as an indicator of water treatment efficacy but are not useful as a definitive indicator of fecal contamination [5]. Project findings will inform education and training to reduce waterborne diseases among PCVs.

## Methods

### Site selection

A convenience sample of PCV households in the Western Highlands of Guatemala was selected to include a variety of site (urban/rural, population) and PCV characteristics (length of service, work project). Travel logistics influenced which PCVs were visited each day.

### Survey

Participants were informed of the date and time that coordinators would visit, but were not given advance notice that water sampling would take place to avoid biasing usual practices. Each PCV was provided an overview of the aims of the project; provided signed informed consent; and responded to a 36-item questionnaire on water types, household water treatment and storage practices, and history of GI illness (defined as “gastrointestinal health problems or diarrhea during service”). A visual validation of sanitation facilities and handwashing locations was conducted.

Primary drinking water was defined as the water type most frequently consumed by the PCV while in the household. Secondary water was defined as other water types available in the household that either served as back-up drinking water when primary drinking water was unavailable or was not intended for drinking. A *tambo* is a 5-gal (18.9-L) bottle containing commercially-purified water; the bottle is often inverted into a plastic base dispenser that has a flip valve. Water filtered and bottled at a location outside the PCV’s household refers to 5-gal bottles filled with piped water that had been filtered at a house or church and resold within the community. A *pila* is an open-air concrete water storage tank.

Data analysis was performed in Microsoft Excel (Redmond, WA) to produce descriptive statistics. To explore differences in self-reported GI illness by water source, a negative binomial regression model was used accounting for overdispersion and using the months of service as an offset, using SAS Studio, release 3.71 (SAS Institute Inc., Cary, NC).

### Water quality sampling and testing

Duplicate samples of primary drinking water and secondary water were collected. FCR was measured in one sample using a Hach® Pocket Colorimeter II (Loveland, CO, USA), according to manufacturer’s instructions. The second sample was aseptically collected in a 100 mL sterile bottle containing sodium thiosulfate (IDEXX, Westbrook, ME, USA) for microbiological analysis. Bottles were stored in a cooler containing ice during transport to a laboratory (average 8.5 h, range 3–14.5 h). Samples were tested for the presence of total coliforms and *E. coli* using the IDEXX Colilert®-18 methodology, according to manufacturer’s instructions. A field blank (distilled water) and positive control (distilled water seeded with fecal waste) were analyzed each day.

## Results

### Survey

In September 2016, 39 PCVs were visited in their host communities, representing 50% of actively serving PCVs in Guatemala at that time. All PCVs contacted agreed to participate. The mean age of PCVs was 27 (range 22–61) years and 26 (70%) PCVs were female. The mean length of time the PCVs had been in Guatemala, including training, was 14.7 (range 1–25) months. All PCVs had a flush/pour-flush toilet and a fixed handwashing location with water and a cleansing agent available (Table 1).

**Table 1:** Demographics, Drinking Water Types, and Gastrointestinal Illness Reported among Surveyed Peace Corps Volunteers in Guatemala, September, 2016

Characteristic	Number (%) of Participants
Sex	
Female	26 (70%)
Male	13 (30%)
Age	
20–25	27 (69%)
26–30	9 (23%)
> 30	3 (8%)
Months of Service	
1–4	2 (5%)
5–8	6 (15%)
9–12	16 (41%)
> 12	15 (38%)
Handwashing location	
Fixed location	39 (100%)
Water available	39 (100%)
Cleansing agent available	39 (100%)
Sanitation facility	
Flush/pour-flush toilet	39 (100%)
Primary drinking water type consumed	
*Tambo*	25 (64%)
Piped water filtered and stored in the household	6 (15%)
Piped water boiled and stored in the household	3 (8%)
Single-use sealed bags of purified water	2 (5%)
Water filtered and bottled at a location outside the PCV’s household	2 (5%)
Piped water treated with chlorine and stored in the household	1 (3%)
Primary drinking water type unavailable for ≥1 day in past 2 weeks	
No	31 (79%)
Yes	8 (21%)
Days per typical week consuming water other than primary drinking water	
0	10 (26%)
1–2	10 (26%)
3–4	11 (28%)
5–7	8 (21%)
Ever had a gastrointestinal illness during service	
Yes	38 (97%)
No	1 (3%)
Number of gastrointestinal illnesses per year of service (*n* = 38)	
1–5	18 (47%)
6–10	8 (21%)
11–15	7 (18%)
16–20	2 (5%)
> 20	3 (8%)

Note: numbers may not add to 100% due to rounding

*Tambo* was the water type most frequently reported as primary drinking water (*n* = 25, 64%), followed by piped water filtered and stored in the household (*n* = 6, 15%), piped water boiled and stored in the household (*n* = 3, 8%), single-use sealed bags (*n* = 2, 5%), water filtered and bottled at a location outside the PCV’s household (*n* = 2, 5%), and piped water treated with chlorine and stored in the household (*n* = 1, 3%). The 10 (26%) PCVs who treated piped water by filtering, boiling, or chlorinating reported this was the only water treatment method used. Approximately one-fifth of PCVs (*n* = 8, 21%) indicated that their primary drinking water type had not been available for one or more days in the past 2 weeks. Three-quarters of PCVs reported that they consume water other than their primary drinking water one or more days per week, with eight (21%) reporting consumption 5–7 days per week.

Nearly all PCVs (*n* = 38, 97%) reported having GI illness at least once during their service. PCVs experienced an average of 1.4 GI illness per PCV per month of service (95% CI [0.9–2.2]). The incidence rate of GI illness per PCV per month of service among PCVs who reported never consuming water other than primary drinking water (0.4, 95% CI [0.1–1.4]) was significantly lower than among PCVs who reported consuming water other than primary drinking water (1.6, 95% CI [1.0–2.5]) (*p* < 0.05).

### Primary drinking water quality

Overall, 37 primary drinking water samples were tested. Most of these were collected from *tambos* (*n* = 23, 62%), followed by piped water filtered and stored in the household (*n* = 6, 16%), piped water boiled and stored in the household (*n* = 2, 5%), single-use sealed bags (*n* = 2, 5%), water filtered and bottled at a location outside the PCV’s household (*n* = 2, 5%), piped water boiled then filtered and stored in the household (*n* = 1, 3%), and piped water treated with chlorine and stored in the household (*n* = 1, 3%) (Table 2).

**Table 2:** Primary Drinking Water and Secondary Water Quality Test Results, Guatemala, September, 2016

Type of water sample	Number (%) Samples Containing < 0.2 mg/L FCR	Number (%) Samples Containing *E. coli*	Number (%) Samples Containing Total Coliforms
Primary drinking water (*n* = 37)	36 (97%)	0 (0%)	25 (68%)
*Tambo* (*n* = 23, 62%)	23 (100%)	0 (0%)	17 (74%)
Single-use sealed bags of purified water (*n* = 2, 5%)	2 (100%)	0 (0%)	0 (0%)
Water filtered and bottled at a location outside the PCV’s household (*n* = 2, 5%)	2 (100%)	0 (0%)	2 (100%)
Piped water filtered and stored in the household (*n* = 6, 16%)	6 (100%)	0 (0%)	4 (67%)
Piped water boiled and stored in the household (*n* = 2, 5%)	2 (100%)	0 (0%)	1 (50%)
Piped water boiled then filtered and stored in the household (n = 1, 3%)	1 (100%)	0 (0%)	0 (0%)
Piped water treated with chlorine and stored in the household (*n* = 1, 3%)	0 (0%)	0 (0%)	1 (100%)^*^
Secondary water (*n* = 43)	40 (93%)	15 (35%)	31 (72%)
Piped water collected directly from the tap (*n* = 34, 79%)	31 (91%)	14 (41%)	25 (74%)
Protected dug well water stored in a rooftop tank, no additional treatment (*n* = 1, 2%)	1 (100%)	1 (100%)	1 (100%)
Tanker truck and rain water mix stored in a *pila*, no additional treatment (*n* = 1, 2%)	1 (100%)	0 (0%)	1 (100%)
Single-use sealed bags of purified water (*n* = 1, 2%)	1 (100%)	0 (0%)	0 (0%)
Water filtered and bottled at a location outside the PCV’s household (*n* = 1, 2%)	1 (100%)	0 (0%)	1 (100%)
Piped water filtered and stored in the household (*n* = 3, 7%)	3 (100%)	0 (0%)	2 (67%)
Piped water boiled and stored in the household (*n* = 2, 5%)	2 (100%)	0 (0%)	1 (50%)
Total (*n* = 80)	76 (95%)	15 (19%)	56 (70%)

^*^Occurrence of total coliforms in water with a free chlorine residual suggests contamination of sample post-collection (after chlorine had been quenched) or survival of microbes due to protection in biofilms that had sloughed off from distribution system pipes or were present on storage container surfaces [19]

Nearly all primary drinking water samples (*n* = 36, 97%) had an FCR < 0.2 mg/L. *E. coli* was not detected in any primary drinking water sample. Total coliforms were detected in 25 (68%) samples: 17/23 (74%) *tambo* samples; 4/6 (67%) samples of piped water filtered and stored in the household; 1/2 (50%) samples of piped water boiled and stored in the household; and 2/2 (100%) samples of water filtered and bottled at a location outside the PCV’s household. One sample of piped water treated with chlorine and stored in the household had an FCR of 1.97 mg/L and was positive for total coliforms.

### Secondary water quality

Overall, 43 secondary water samples were tested. Most of these were piped water collected directly from the tap (i.e., did not receive any additional treatment in the household) (*n* = 34, 79%), followed by piped water filtered and stored in the household (*n* = 3, 7%), piped water boiled and stored in the household (*n* = 2, 5%), single-use sealed bags (*n* = 1, 2%); protected dug well water stored in a rooftop tank (*n* = 1, 2%), water filtered and bottled at a location outside the PCV’s household (*n* = 1, 2%), and a mix of tanker truck and rain water stored in a *pila* (*n* = 1, 2%).

Nearly all secondary water samples (*n* = 40, 93%) had an FCR < 0.2 mg/L; however, three (9%) piped water samples collected directly from the tap had FCR concentrations of 0.21, 0.40, and 0.64 mg/L, respectively. *E. coli* was detected in 15 (35%) secondary water samples: 14/34 (41%) piped water samples collected directly from the tap and 1/1 (100%) sample originating from a protected dug well and stored in a rooftop tank. Total coliforms were detected in 31 (72%) samples: 25/34 (74%) piped water samples collected directly from the tap, 2/3 (67%) samples of piped water filtered and stored in the household, 1/2 (50%) samples of piped water boiled and stored in the household, 1/1 (100%) sample originating from a protected dug well and stored in a rooftop tank, 1/1 (100%) sample of water filtered and bottled at a location outside the PCV’s household, and 1/1 (100%) sample of a mix of tanker truck and rain water stored in a *pila*.

## Discussion

Over two-thirds of Peace Corps Volunteers in Guatemala reported their most frequently consumed water type in the household was commercially-purified water (*tambos* or single-use sealed bags) and more than 25% reported consuming piped water that they had boiled, filtered, or chlorinated. All primary drinking water samples tested met the WHO recommendation of < 1 *E. coli* per 100 mL [5]. While nearly all PCVs reported having experienced GI illness during their service, the rate of GI illness among PCVs who reported never consuming water other than primary drinking water was significantly lower than among PCVs who reported consuming other water types at least once per week. These findings suggest that consuming primary drinking water exclusively likely contributes to reducing the rate of GI illness among PCVs.

Twenty-one percent of PCVs indicated that their primary drinking water type had not been available for one or more days in the past 2 weeks and 74% indicated they consumed water other than their primary drinking water one or more days per week. While these other water types were not specifically investigated, other water types present in the household, largely piped water collected directly from the tap, were tested. More than one-third of all secondary water samples tested contained *E. coli* and nearly all (93%) had an FCR < 0.2 mg/L, supporting a previous finding that few municipalities in Guatemala provide adequately chlorinated water to their communities as required by Guatemala’s Municipal Code [6]. While most secondary water was not necessarily intended for consumption, direct or indirect (e.g., using water for uncooked food preparation) consumption when primary water types are unavailable or when outside the household, may put PCVs at risk for GI illness if these water types are not properly treated and stored.

Chlorine is highly effective in inactivating most microorganisms and offers the benefit of maintaining a residual in water that protects against recontamination [7]. Treating water with chlorine has been shown to reduce self-reported diarrheal disease [8]. WHO recommends a free chlorine concentration of 0.2 mg/L [5]; when water is dosed properly, this concentration can be maintained for up to 24 h [9, 10]. The majority (97%) of primary drinking water samples tested in PCV households had an FCR < 0.2 mg/L. This is expected in water that had been commercially-purified by processes that do not result in a chlorine residual in the final product (e.g., reverse osmosis, ultraviolet light irradiation). A quarter of PCVs reported their primary drinking water type was piped water treated in the household, however filtration and boiling can reduce or remove chlorine and chlorine manually added to water can naturally decay over time. Stored drinking water that does not contain an adequate disinfectant residual is vulnerable to microbial contamination. Indeed, two-thirds of primary drinking water samples contained total coliforms, suggesting inadequate water treatment or recontamination after treatment. Researchers have recognized the difficulty that public health organizations face in the real-world implementation of water retreatment every 24 h [9]. Recontamination can be reduced by storing water in containers with small openings and taps that prevent users from introducing potentially contaminated items into the stored water and by regularly cleaning storage containers [10].

Studies of short-term travelers, journalists, and relief workers have reported that GI illness is common among these populations (28–80% of individuals) [11–17]. In many cases, increased duration of travel was correlated with decreased adherence to food and water hygiene recommendations [11] and with increased GI illness [11–13]. As PCVs commit to service for over two years, training PCVs on safe food and water preparation is important; studies have shown pre-travel health consultation correlates with decreased risk of GI illness [11, 14].

This project was subject to several limitations. First, its small sample size, especially in subcategories of water types and the availability of water at the time of the visit, prevented a more thorough assessment of water types consumed in PCV households. Second, the focus of this project was primary drinking water, but further insights into other sources of GI risks PCVs face could be gained by assessing water consumed outside of PCV households and PCV behaviors when primary drinking water is not available, as well as food exposures. For example, one study found that beverages prepared using tap water and sold from market vendors in Guatemala City were contaminated with fecal coliform bacteria (44–52%) and *E. coli* (15–19%) [18]. Third, water samples were collected at one point in time and may not reflect water quality in households over time or account for seasonal variation. Fourth, GI illness counts were self-reported estimates without clinical or lab confirmation, so it was not possible to differentiate between GI symptoms and GI illness due to infectious agents. Lastly, handwashing and food preparation and hygiene behaviors were not addressed; assessing these may reveal other factors that contribute to GI illness.

## Conclusions

To conclude, consuming primary drinking water exclusively likely contributes to reducing the rate of GI illness among PCVs, as demonstrated by the finding that the rate of GI illness among PCVs who reported never consuming water other than primary drinking water was significantly lower than among PCVs who reported consuming other water types at least once per week. The water quality and behavioral findings of this project will be used to inform education and training for the purpose of reducing waterborne diseases among PCVs. The findings support existing recommendations such as only consuming water that has been treated using Peace Corps-recommended water methods, even when outside the home, as well as following guidelines for safe storage of water in the household. These findings and recommendations may be useful for naïve expatriate workers living in community settings in developing countries, however, PCVs are a unique population in terms of protective and risk factors and types of exposures, and the results of this project may not be readily generalizable to other populations.

## Abbreviations

CDC: Centers for Disease Control and Prevention
*E. coli*: *Escherichia coli*
FCR: Free Chlorine Residual
GI: Gastrointestinal
PCV: Peace Corps Volunteer
WHO: World Health Organization

## References

[1] Peace Corps. The Peace Corps Mission [internet]. Washington DC https://www.peacecorps.gov/about/. Accessed 25 Oct 2016.

[2] Peace Corps Office of Medical Services. The Health of the Volunteer 2016. Annual Report of Volunteer Health. Washington DC. Peace Corps Office of Medical Services, Epidemiology and Surveillance Unit. 2017.

[3] Peace Corps. Preparation & Training [internet]. Washington DC. https://www.peacecorps.gov/volunteer/preparation-and-training/. Accessed 25 Oct 2016.

[4] Peace Corps Guatemala. 2016. Summary of water disinfection methods. [Personal communication 2016 Oct 19].

[5] World Health Organization (WHO). World Health Organization Guidelines for drinking-water quality, 4th edition. 2011. Geneva, World Health Organization. http://apps.who.int/iris/bitstream/10665/44584/1/9789241548151_eng.pdf. Accessed 25 Oct 2016.

[6] Avila C, Bright R, Gutierrez J, Hoadley K, Manuel C, Romero N, et al. Guatemala Health System Assessment, August 2015. Bethesda, MD: Health Finance & Governance Project, Abt Associates Inc. https://www.usaid.gov/sites/default/files/documents/1862/Guatemala-HSA%20_ENG-FULL-REPORT-FINAL-APRIL-2016.pdf. Accessed 25 Oct 2016.

[7] Branz A, Levine M, Lehmann L, Bastable A, Ali SI, Kadir K (2017). Chlorination of drinking water in emergencies: a review of knowledge to develop recommendations for implementation and research needed. Waterlines.

[8] Arnold BF, Colford JM (2007). Treating water with chlorine at point-of-use to improve water quality and reduce child diarrhea in developing countries: a systematic review and meta-analysis. Am J Trop Med Hyg.

[9] Null C, Lantagne D (2012). Microbiological quality of chlorinated water after storage in ceramic pots. J Water Sanit Hyg Dev.

[10] Wilhelm N, Kaufmann A, Blanton E, Lantagne D (2018). Sodium hypochlorite dosage for household and emergency water treatment: updated recommendations. J Water Health.

[11] Mattila L, Siitonen A, Kyrönseppä H, Simula I, Peltola H (1995). Risk behavior for travelers’ diarrhea among Finnish travelers. J Travel Med.

[12] Hill DR (2000). Health problems in a large cohort of Americans traveling to developing countries. J Travel Med.

[13] Winer L, Alkan M (2002). Incidence and precipitating factors of morbidity among Israeli travelers abroad. J Travel Med.

[14] Bhatta P, Simkhada P, Van Teijlingen E, Maybin S (2009). A questionnaire study of voluntary service overseas (VSO) volunteers: health risk and problems encountered. J Travel Med.

[15] Sharp TW, DeFraites RF, Thornton SA, Burans J, Wallace M (1995). Illness in journalists and relief workers involved in international humanitarian assistance efforts in Somalia, 1992–93. J Travel Med.

[16] Harvey K, Esposito DH, Han P, Kozarsky P, Freedman D, Plier D (2013). Surveillance for travel-related disease—GeoSentinel surveillance system, United States, 1997–2011. MMWR Surveill Summ.

[17] Kunin SB, Kanze DM (2016). Care for the Health Care Provider. Med Clin North Am.

[18] Sobel J, Mahon B, Mendoza C, Passaro C, Cano F, Baier K (1998). Reduction of fecal contamination of street-vended beverages in Guatemala by a simple system for water purification and storage, handwashing, and beverage storage. Am J Trop Med Hyg.

[19] Jagals P, Jagals C, Bokako TC (2003). The effect of container-biofilm on the microbiological quality of water used from plastic household containers. J Water Health.

